# The Role of Covered Stents in Hemodialysis Access: Experience From a Vascular Access Centre

**DOI:** 10.7759/cureus.81496

**Published:** 2025-03-31

**Authors:** Andreia Henriques, João Venda, Emanuel Ferreira, Nuno A Oliveira

**Affiliations:** 1 Nephrology, Centro Hospitalar e Universitário de Coimbra, Coimbra, PRT; 2 Interventional Nephrology, Centro de Acesso Vascular SANFIL, Coimbra, PRT

**Keywords:** angioplasty, arteriovenous fistula, arteriovenous graft, covered stent, hemodialysis

## Abstract

Background

Vascular access (VA) dysfunction in hemodialysis (HD) significantly impacts patient outcomes. While percutaneous transluminal angioplasty remains the primary intervention, covered stents (CSs) have emerged as a valuable adjunct. This study evaluates the efficacy of CS in reducing reinterventions and delaying subsequent procedures.

Methods

This retrospective study included patients who underwent their first CS placement at the SANFIL Vascular Access Centre between 2017 and 2022, involving arteriovenous fistulas (AVFs) and arteriovenous grafts (AVGs). The primary outcome was a comparison of the number of procedures 12 months before and after CS placement. Secondary outcomes assessed primary patency (PP), assisted primary patency (APP), secondary patency (SP), and factors influencing these outcomes.

Results

Eleven patients were included, 72.7% male, with a mean age of 74.6 ± 8.8 years. Seven (63.6%) patients had an AVF. In the 12 months after CS placement, only two VAs did not require reintervention, while the remaining nine exhibited CS-related dysfunctions. The mean number of interventions in the 12 months before and after CS placement was similar (1.73 ± 1.01 and 1.64 ± 1.63, respectively; p = 0.85). However, the mean time to reintervention after CS placement was longer than the previous intervention: 6.22 ± 3.67 and 2.63 ± 2.60 months, respectively. PP was significantly higher in AVFs than in AVGs.

Conclusions

CS placement may delay the need for subsequent interventions, but it does not significantly reduce the frequency of procedures or improve overall VA survival. The decision to deploy a CS should be made on a case-by-case basis, particularly for patients with limited vascular options or those who are unable to undergo additional surgical procedures. Careful patient selection is essential to ensure the optimal use of CS in HD patients.

## Introduction

A reliable vascular access (VA) is essential for effective hemodialysis (HD). Current recommendations endorse autogenous arteriovenous fistulas (AVFs) and arteriovenous grafts (AVGs) as the preferred types of VA for HD [1]. However, VAs are prone to dysfunction, often developing stenosis and subsequent thrombosis. VA dysfunction significantly contributes to morbidity and mortality among HD patients and incurs substantial economic costs [2,3]. Consequently, there is an ongoing search for more effective therapeutic strategies. Percutaneous transluminal angioplasty (PTA) is the primary therapeutic approach, yielding satisfactory initial results [1]. However, it often faces challenges, including frequent and early restenosis, recoil, and PTA-induced rupture. These complications typically require multiple additional procedures and ultimately result in VA loss [2,4]. To address these issues, stents, particularly covered stents (CSs), have emerged as a promising alternative. CSs are self-expanding bare-metal stents covered on either the internal or external surface with a material, most commonly expanded polytetrafluoroethylene (ePTFE) [5]. These stents are believed to maintain patency by exerting a sustained mechanical force, reducing turbulence through laminar flow, and creating a barrier between the endothelium and the bloodstream [4-7]. Initially, bare-metal stents were applied, but their use was associated with frequent in-stent restenosis caused by tissue ingrowth resulting from smooth muscle cell migration and neovascularization. In response, stents featuring a covering material were developed to inhibit tissue ingrowth and neointimal hyperplasia, thereby reducing restenosis [8,9]. Currently, CSs are indicated for vascular rupture, residual stenosis/recoil exceeding 30% after PTA, or recurrent stenoses within three months [1,10-12]. However, using CS increases procedural costs and may introduce potential complications.

This study aims to assess the impact of CS placement on VA outcomes in HD patients by evaluating its effect on the frequency of reinterventions, time to subsequent interventions, and patency rates.

## Materials and methods

This retrospective cohort study included individuals aged 18 years and older who underwent their first CS placement, either in AVFs or AVGs, at a Vascular Access Centre between 2017 and 2022. Interventional nephrologists performed the CS placement, with the same team (two nephrologists) being involved in the CS deployment. Data were collected from the Vascular Access Centre database. A comparative analysis was conducted, assessing the 12-month periods preceding and following the CS placement. Patients were excluded if they had their VA for less than 12 months before the first intervention, were lost to follow-up during the study period, or had undergone bare-metal stent placement.

The primary outcome was the comparison of the number of procedures conducted in the 12 months before and after the CS placement. Secondary outcomes included primary patency (PP), defined as the time from CS placement to the first intervention, thrombosis, or VA abandonment; assisted primary patency (APP), defined as the time from CS placement to the first occurrence of thrombosis or VA abandonment; and secondary patency (SP), defined as the time from CS placement to VA abandonment. Furthermore, clinical and demographic factors influencing the number of procedures, PP, APP, and SP were also evaluated.

The analysis was performed using IBM SPSS Statistics for Windows, Version 20 (Released 2011; IBM Corp., Armonk, NY, USA), employing paired t-tests to compare intervention rates before and after CS placement, alongside Kaplan-Meier survival analyses to assess patency outcomes. For multivariate analysis, Cox proportional hazards models and mixed-effects regression models were utilized.

The study was conducted in accordance with the Declaration of Helsinki, and informed consent for anonymized data collection was obtained from all participants.

## Results

A total of 20 patients underwent stent placement, of whom 11 were included in the study. Among these 11 patients, 72.7% (n = 8) were male, with a mean age of 74.6 ± 8.8 years. Of these patients, 36.4% (n = 4) had an AVG, and 63.6% (n = 7) had an AVF, with a mean VA duration of 33.8 ± 19.8 months. The duration of HD exceeded five years in 63.6% (n = 7) of patients. Our study included patients of advanced age (mean age 74.6 ± 8.8 years), with 36.4% having an AVG and 45.5% having diabetes. Detailed demographic characteristics, VA type, etiology of chronic kidney disease, and comorbidities are presented in Table 1.

**Table 1:** Characteristics of patients that underwent covered stent placement.

Demographics	
Men, n (%)	8 (72.7%)
Arteriovenous graft, n (%)	4 (36.4%)
Mean age of onset of hemodialysis, years	69.0 ± 9.2
Mean time on hemodialysis, years	5.7 ± 3.6
Mean age at the time of covered stent placement, years	74.6 ± 8.8
Mean lifetime of the arteriovenous access, months	33.8 ± 19.8
Vascular Access	
Brachial-axillary arteriovenous graft, n (%)	4 (26.4%)
Brachial-basilic arteriovenous fistula, n (%)	3 (27.3%)
Brachial-cephalic arteriovenous fistula, n (%)	3 (27.3%)
Radio-cephalic arteriovenous fistula, n (%)	1 (9.1%)
Etiology of Chronic Kidney Disease	
Diabetes, n (%)	5 (45.5%)
Chronic glomerulonephritis, n (%)	2 (18.2%)
Unknown, n (%)	2 (18.2%)
Polycystic kidney disease, n (%)	1 (9.1%)
Other, n (%)	1 (9.1%)
Comorbidities	
Hypertension, n (%)	10 (90.9%)
Diabetes, n (%)	5 (45.5%)
Dyslipidemia, n (%)	10 (90.9%)
Body mass index > 25 Kg/m^2^, n (%)	2 (18.2%)
Smoking history, n (%)	0
Heart failure with reduced ejection fraction, n (%)	2 (18.2%)
Coronary artery disease, n (%)	1 (9.1%)
Peripheral artery disease, n (%)	2 (18.2%)
Implantable cardioverter-defibrillator/Pacemaker/Implantofix®, n (%)	1 (9.1%)
Antiplatelets, n (%)	4 (36.4%)
Anticoagulants, n (%)	5 (45.5%)
Embolism (stroke/pulmonary embolism/deep vein thrombosis), n (%)	2 (18.2%)
Atrial fibrillation, n (%)	2 (18.2%)

The CS utilized included the Covera® Stent Graft (Becton, Dickinson and Company, Franklin Lakes, NJ, USA) in 50.0% (n = 5) of cases, the Fluency Plus® Stent (Becton, Dickinson and Company) in 7.1% (n = 1) of cases, and CSs of unspecified brands in the remaining cases. The dimensions of the utilized CSs were 9 x 40 mm in 78.6% (n = 8) of patients, and 10 x 40 mm in 21.4% (n = 3) of patients.

CSs were placed in outflow stenoses in all cases: in the axillary vein in five (45.5%) cases, in the transition from the axillary vein to the subclavian vein in two (18.2%) cases, in the cephalic vein of the arm in two (18.2%) cases, and at the junction between the graft and the axillary vein in two (18.2%) cases. In 45.5% (n = 5) of the patients, the VAs were thrombosed at the start of the procedure of CS placement, with all such cases involving AVFs. The most common reason for CS placement was early recurrent stenosis (n = 6, or 54.5%), followed by residual stenosis/recoil exceeding 30% after PTA (n = 3, or 27.3%). One patient presented with both early recurrence and residual stenosis exceeding 30%. Additional reasons for CS placement included a history of multiple prior interventions (n = 2, or 18.2%) and uncontrolled vascular rupture by endoluminal tamponade (n = 1, or 9.1%).

**Figure 1:**
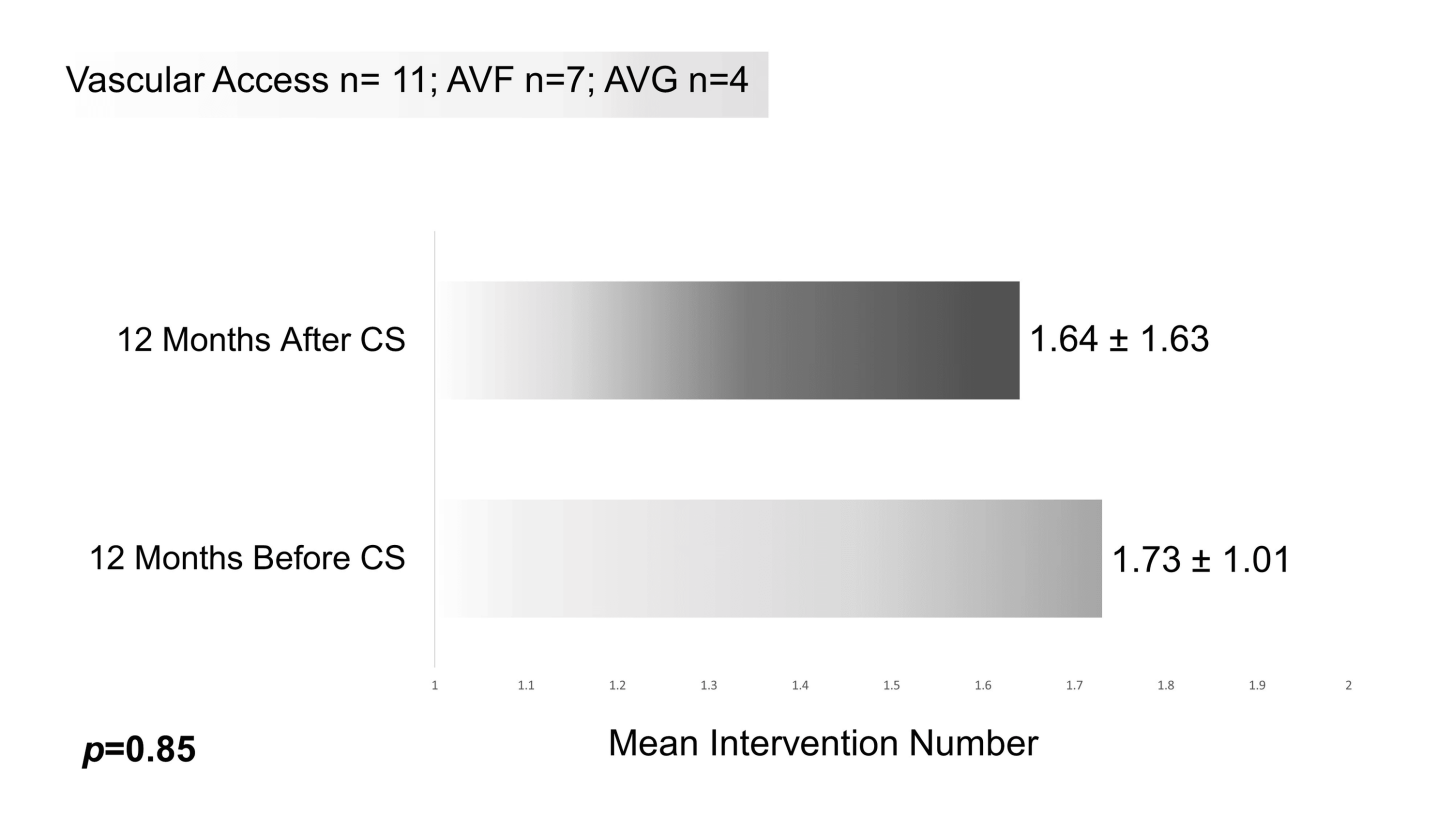
Mean intervention number in the 12 months preceding and following CS placement. AVF, arteriovenous fistula; AVG, arteriovenous graft; CS, covered stent

In the lifetime of the VA before CS placement, all VAs had already been intervened upon, except one, with a mean number of procedures of 2.45 ± 1.64. In the 12 months before and after CS placement, the mean number of procedures was 1.73 ± 1.01 and 1.64 ± 1.63, respectively, revealing no statistically significant difference (T(10) = 0.20, p = 0.85) (Figure 1). The number of procedures following CS placement did not differ significantly between AVGs and AVFs (T(3.41) = -2.14, p = 0.12). Similarly, there was no statistically significant difference in the number of procedures between thrombosed VAs at the start of the procedure and those that were not (T(9) = 1.69, p = 0.13).

**Figure 2:**
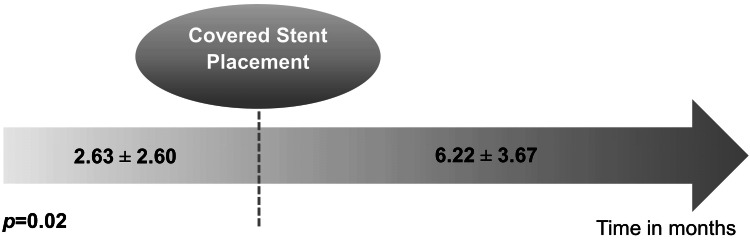
Mean time between the last intervention before covered stent placement and the first intervention after covered placement.

Regarding the intervention time, the previous procedure before CS placement was, on average, 2.63 ± 2.60 months prior, and the mean time to reintervention after CS placement was 6.22 ± 3.67 months, showing a statistically significant difference (T(7) = -2.95, p = 0.02) (Figure 2).

A multivariate analysis (linear regression) was conducted, which included variables such as sex, age, and patient comorbidities (including cardiovascular factors). The analysis revealed no statistically significant influence of these factors on the time to reintervention after CS placement. Two VAs did not require reintervention after CS placement. Among the VAs that did require reintervention, all presented with stenosis at the CS site, with all cases exhibiting stenosis at the CS ends. One (11.11%) VA displayed both stenosis within the CS and at its end. Only one VA was abandoned due to thrombosis at four months.

The data for PP, APP, and SP at three, six, and twelve months after CS placement are detailed in Table 2, encompassing both the overall VAs and categories separated by AVFs and AVGs. The PP was significantly higher in AVFs compared to AVGs (HR = 4.84; 95% CI = 1.05, 22.27; p = 0.043), a finding that remained significant after performing multivariate analysis, which adjusted for cardiovascular factors, patient age, and sex. The analysis revealed no statistically significant association between cardiovascular risk factors, including blood pressure, diabetes, dyslipidemia, body mass index, and smoking habits, with the number of interventions, PP, APP, and SP. Similarly, variables such as sex, the use of antiplatelets or anticoagulants, and previous thromboses or thrombosis at the time of CS placement did not show any significant influence.

**Table 2:** Mean time until first procedure, primary patency, assisted primary patency and the secondary patency at 3, 6 and 12 months, for all VA and for AVFs and AVGs. AVF, arteriovenous fistula; AVG, arteriovenous graft; VA, vascular access

	Mean Time Until 1st Procedure (Months)	p-value	No. of Patent VAs at 3 Months	No. of Patent VAs at 6 Months	No. of Patent VAs at 12 Months
Primary Patency					
Total VA (11)	6.2 ± 2.6		9 (81.8%)	6 (54.6%)	2 (18.2%)
AVF (7)	8.0 ± 3.8	0.043	7 (100%)	5 (71.4%)	2 (28.6%)
AVG (4)	4.0 ± 2.2		2 (50%)	1 (25%)	0
Assisted Primary Patency					
Total VA (11)	8.33 ± 4.1		11 (100%)	8 (72.7%)	5 (45.5%)
AVF (7)	9.33 ± 4.6	0.244	7 (100%)	1 (14.3%)	4 (57.1%)
AVG (4)	7.33 ± 4.2		4 (100%)	2 (50%)	1 (25%)
Secondary Patency					
Total VA (11)	4 ± 0		11 (100%)	10 (90.9%)	10 (90.9%)
AVF (7)	4 ± 0	0.450	7 (100%)	6 (85.7%)	6 (85.7%)
AVG (4)	-		4 (100%)	4 (100%)	4 (100%)

## Discussion

Despite the growing popularity of CS as an adjunct to conventional interventions such as PTA, our study did not reveal a statistically significant difference in the number of procedures performed within the 12 months before and after CS placement. Previous studies have suggested that CS may reduce the need for reinterventions in VAs compared to PTA alone [6,7,13]. However, it is crucial to note that these studies compared distinct patient groups - those subjected to PTA alone and those undergoing CS placement - whereas our investigation assessed the same VA both before and after CS placement, providing a unique perspective.

The patient population in this study consisted primarily of elderly patients; 45.5% had diabetes, with a high proportion of these having AVGs (36.4%) and longstanding VA dysfunction (mean VA duration: 33.8 ± 19.8 months). Notably, these are factors known to be associated with worse VA outcomes and higher intervention rates [14]. Nearly all VAs in our study had undergone prior procedures, several more than once, and often treated simultaneously for several stenoses. Managing multiple stenoses amplifies potential sites for restenosis. Understandably, VAs that have undergone multiple interventions - whether PTA alone or with CS placement - carry a heightened risk of developing new stenoses, recurring previous stenosis, or VA thrombosis, which can decrease VA circuit patency [7]. Our analysis reveals that CS may not effectively reduce the number of procedures needed for problematic VAs.

In our study, most VAs developed stenoses at the ends of CSs, which was consistent with prior reports indicating that a significant proportion of stenosis recurrences occur outside the target lesion area with CSs. Unlike bare-metal stents, where stenosis recurrences typically occur within the stent, CSs tend to exhibit recurrent stenosis at their ends, along the edges of the implanted stent [9,15].

The most common reason for CS placement in our study was rapidly recurrent stenosis, a recognized limitation of PTA alone, with PP ranging from 20% to 50% within six months [16]. Literature reports lower PP when CSs are applied near the arterial anastomotic region.

We found a six-month PP of 54.6%, which aligns with existing literature, including more recent studies [17]. Various trials, including FLAIR® Pivotal [18], REVISE [15], and RENOVA [19], have shown that CS placement leads to higher PP compared to PTA alone. The AVeNEW trial, focusing on AVF stenosis, showed increased target lesion patency with CSs, though no significant difference in overall circuit patency over 24 months was noted [7]. Notably, a systematic review also echoed a trend towards improved PP at 6 and 12 months with CSs, but no statistically significant difference at 24 months [6]. Our study demonstrated a significant prolongation in the time to reintervention after CS placement (mean: 6.22 ± 3.67 months), compared to the interval before CS deployment (mean: 2.63 ± 2.60 months, p = 0.02). This suggests that CS may contribute to delaying restenosis or VA failure, potentially improving short-term VA function. However, although CS may offer higher initial PP, this benefit appears to diminish over time, potentially resulting in no overall advantage in VA survival [9,16].

Consistent with previous reports, our study revealed a superior PP with CS placement in AVFs compared to AVGs [20,21]. Some studies attributed this difference to the increased propensity of AVGs to present with thrombosis at the time of CS placement, which negatively impacts VA patency [15,20,21]. However, due to the limited sample size, our analysis was unable to identify a significant difference in PP between VAs that were thrombosed at the time of CS placement and those that were not. Therefore, the higher PP observed in AVFs may be explained by the inherent characteristics of these accesses, which generally exhibit better PP compared to AVGs, irrespective of CS utilization [14].

Lastly, a pivotal consideration regarding the use of CS lies in the financial aspect. The economic value of CS placement compared to PTA alone remains uncertain [13,22,23]. While CS placement involves higher initial costs, it is expected that this cost might be offset by a reduction in the number of reinterventions required to maintain VA patency. Our study did not conduct a comprehensive cost assessment, but the lack of a significant reduction in the number of reinterventions suggests that CS may not be cost-effective. Future studies, incorporating cost-effectiveness analyses, could further strengthen this aspect.

It is important to acknowledge the limitations of our study. The relatively small sample size and retrospective design may affect the generalizability of our findings. These constraints highlight the need for larger, prospective studies to validate our results and assess their broader applicability.

## Conclusions

The placement of CS may extend the time until subsequent interventions, but does not necessarily reduce the frequency of procedures required for VA maintenance or improve overall VA survival. These findings suggest that CS may not be considered a routine alternative to PTA, but rather a selective intervention for specific cases. We believe that this approach may be particularly relevant for patients with exhausted vascular capital, for older and fragile patients who may not tolerate another surgical procedure for AVF creation, and for those aiming to preserve one of the patient's last viable VAs. Therefore, the decision to deploy a CS should be made on a case-by-case basis, considering the specific clinical circumstances of each patient.
